# 4-Hydr­oxy-*N*′-(4-methoxy­benzyl­idene)benzohydrazide

**DOI:** 10.1107/S1600536809030621

**Published:** 2009-08-08

**Authors:** Da-Hua Shi

**Affiliations:** a School of Chemical Engineering, Huaihai Institute of Technology, Lianyungang Jiangsu 222005, People’s Republic of China

## Abstract

The title compound, C_15_H_14_N_2_O_3_, was synthesized by the reaction of 4-methoxy­benzaldehyde with 4-hydroxy­benzohydrazide in methanol. The mol­ecule adopts an *E* configuration about the C=N bond. The two benzene rings make a dihedral angle of 46.6 (2)°. In the crystal structure, mol­ecules are linked into a two-dimensional network parallel to (001) through O—H⋯O and N—H⋯O hydrogen bonds.

## Related literature

For the anti­bacterial activity of hydrazone compounds, see: Cukurovali *et al.* (2006 ▶). For crystal structures of hydrazone compounds, see: Abdul Alhadi *et al.* (2009 ▶); Mohd Lair *et al.* (2009 ▶); Cao & Lu (2009 ▶); Qu & Cao (2009 ▶). For bond-length data, see: Allen *et al.* (1987 ▶).
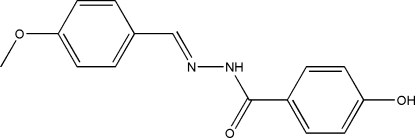


## Experimental

### 

#### Crystal data

C_15_H_14_N_2_O_3_
*M* *_r_* = 270.28
Orthorhombic, 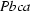

*a* = 11.947 (2) Å
*b* = 7.555 (1) Å
*c* = 29.452 (2) Å
*V* = 2658.3 (6) Å^3^
*Z* = 8
Mo *K*α radiation
μ = 0.10 mm^−1^
*T* = 298 K
0.20 × 0.20 × 0.17 mm

#### Data collection

Bruker SMART CCD area-detector diffractometer
Absorption correction: multi-scan (*SADABS*; Bruker, 2001 ▶) *T*_min_ = 0.981, *T*_max_ = 0.984
15205 measured reflections
2897 independent reflections
2030 reflections with *I* > 2σ(*I*)
*R*_int_ = 0.035

#### Refinement

*R*[*F* ^2^ > 2σ(*F* ^2^)] = 0.038
*wR*(*F* ^2^) = 0.103
*S* = 1.04
2897 reflections
187 parameters
1 restraint
H atoms treated by a mixture of independent and constrained refinement
Δρ_max_ = 0.18 e Å^−3^
Δρ_min_ = −0.15 e Å^−3^

### 

Data collection: *SMART* (Bruker, 2007 ▶); cell refinement: *SAINT* (Bruker, 2007 ▶); data reduction: *SAINT*; program(s) used to solve structure: *SHELXTL* (Sheldrick, 2008 ▶); program(s) used to refine structure: *SHELXTL*; molecular graphics: *SHELXTL*; software used to prepare material for publication: *SHELXTL*.

## Supplementary Material

Crystal structure: contains datablocks global, I. DOI: 10.1107/S1600536809030621/ci2875sup1.cif

Structure factors: contains datablocks I. DOI: 10.1107/S1600536809030621/ci2875Isup2.hkl

Additional supplementary materials:  crystallographic information; 3D view; checkCIF report

## Figures and Tables

**Table 1:** Hydrogen-bond geometry (Å, °)

*D*—H⋯*A*	*D*—H	H⋯*A*	*D*⋯*A*	*D*—H⋯*A*
O3—H3⋯O2^i^	0.82	1.91	2.7271 (15)	171
N2—H2*A*⋯O2^ii^	0.90 (1)	2.12 (1)	3.0216 (17)	173 (2)

Symmetry codes: (i) 
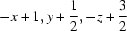
; (ii) 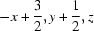.

**Fig. 1.**
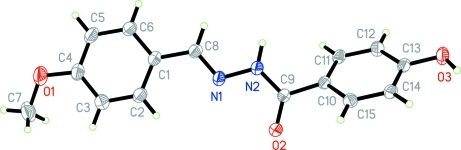
The molecular structure of the title compound. Displacement ellipsoids are drawn at the 30% probability level.

**Fig. 2.**
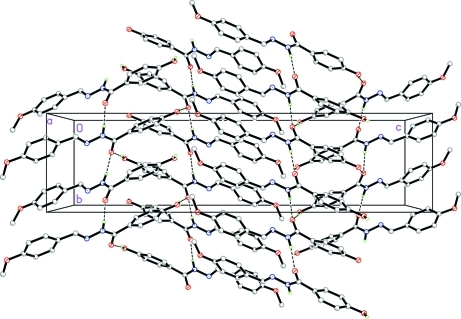
The molecular packing of the title compound, viewed along the a axis. Hydrogen bonds are shown as dashed lines.

## supplementary crystallographic information

### Comment

Hydrazone compounds are a kind of important materials in biological and medicinal chemistry. They have been widely investigated for their antibacterial activities (Cukurovali *et al.*, 2006). Recently, a number of hydrazone compounds have been prepared and structurally characterized (Abdul Alhadi *et al.*, 2009; Mohd Lair *et al.*, 2009; Cao & Lu, 2009; Qu & Cao, 2009). In this paper, the author reports the crystal structure of the title hydrazone compound.

The molecule of the title compound adopts an E configuration about the C═N bond (Fig. 1). The two benzene rings make a dihedral angle of 46.56 (7)°. The C8/N1/N2/C9/O2 plane makes dihedral angles of 8.2 (1) and 54.5 (1)°, respectively, with the C1—C6 and C10—C15 benzene rings. All the bond lengths are normal (Allen *et al.*, 1987).

In the crystal structure, molecules are linked through N—H···O and O—H···O hydrogen bonds (Table 1), forming a two-dimensional network parallel to the (001) [Fig. 2].

### Experimental

4-Methoxybenzaldehyde (13.6 mg, 0.1 mmol) and 4-hydroxybenzohydrazide (15.2 mg, 0.1 mmol) were mixed and refluxed in a methanol solution. After 30 min, the clear solution was evaporated to give colourless crystallites, which were recrystallized from methanol to form single crystals.

### Refinement

Atom H2A was located in a difference Fourier map and refined isotropically, with the N-H distance restrained to 0.90 (1) Å. Other H atoms were placed in calculated positions [O-H = 0.82 Å and C-H = 0.93–0.96 Å] and constrained to ride on their parent atoms, with *U*_iso_(H) = 1.2*U*_eq_(C) and 1.5*U*_eq_(O and C7).

### Crystal data

**Table:**

C_15_H_14_N_2_O_3_	*F*(000) = 1136
*M**_r_* = 270.28	*D*_x_ = 1.351 Mg m^−^^3^
Orthorhombic, *P**b**c**a*	Mo *K*α radiation, λ = 0.71073 Å
Hall symbol: -P 2ac 2ab	Cell parameters from 2853 reflections
*a* = 11.947 (2) Å	θ = 2.6–25.0°
*b* = 7.555 (1) Å	µ = 0.10 mm^−^^1^
*c* = 29.452 (2) Å	*T* = 298 K
*V* = 2658.3 (6) Å^3^	Block, colourless
*Z* = 8	0.20 × 0.20 × 0.17 mm

### Data collection

**Table:**

Bruker SMART CCD area-detector diffractometer	2897 independent reflections
Radiation source: fine-focus sealed tube	2030 reflections with *I* > 2σ(*I*)
graphite	*R*_int_ = 0.035
ω scans	θ_max_ = 27.0°, θ_min_ = 1.4°
Absorption correction: multi-scan (*SADABS*; Bruker, 2001)	*h* = −13→15
*T*_min_ = 0.981, *T*_max_ = 0.984	*k* = −9→9
15205 measured reflections	*l* = −33→37

### Refinement

**Table:**

Refinement on *F*^2^	Secondary atom site location: difference Fourier map
Least-squares matrix: full	Hydrogen site location: inferred from neighbouring sites
*R*[*F*^2^ > 2σ(*F*^2^)] = 0.038	H atoms treated by a mixture of independent and constrained refinement
*wR*(*F*^2^) = 0.103	*w* = 1/[σ^2^(*F*_o_^2^) + (0.0418*P*)^2^ + 0.5702*P*] where *P* = (*F*_o_^2^ + 2*F*_c_^2^)/3
*S* = 1.04	(Δ/σ)_max_ = 0.001
2897 reflections	Δρ_max_ = 0.18 e Å^−^^3^
187 parameters	Δρ_min_ = −0.15 e Å^−^^3^
1 restraint	Extinction correction: *SHELXTL* (Sheldrick, 2008), Fc^*^=kFc[1+0.001xFc^2^λ^3^/sin(2θ)]^-1/4^
Primary atom site location: structure-invariant direct methods	Extinction coefficient: 0.0025 (7)

### Special details

**Table:**

Geometry. All e.s.d.'s (except the e.s.d. in the dihedral angle between two l.s. planes) are estimated using the full covariance matrix. The cell e.s.d.'s are taken into account individually in the estimation of e.s.d.'s in distances, angles and torsion angles; correlations between e.s.d.'s in cell parameters are only used when they are defined by crystal symmetry. An approximate (isotropic) treatment of cell e.s.d.'s is used for estimating e.s.d.'s involving l.s. planes.
Refinement. Refinement of *F*^2^ against ALL reflections. The weighted *R*-factor *wR* and goodness of fit *S* are based on *F*^2^, conventional *R*-factors *R* are based on *F*, with *F* set to zero for negative *F*^2^. The threshold expression of *F*^2^ > σ(*F*^2^) is used only for calculating *R*-factors(gt) *etc*. and is not relevant to the choice of reflections for refinement. *R*-factors based on *F*^2^ are statistically about twice as large as those based on *F*, and *R*- factors based on ALL data will be even larger.

### Fractional atomic coordinates and isotropic or equivalent isotropic displacement parameters (Å^2^)

**Table:**

	*x*	*y*	*z*	*U*_iso_*/*U*_eq_	
N1	0.77001 (10)	0.20495 (17)	0.58903 (4)	0.0428 (3)	
N2	0.75665 (11)	0.28526 (17)	0.63096 (4)	0.0427 (3)	
O1	0.93859 (13)	0.00418 (19)	0.39100 (4)	0.0755 (4)	
O2	0.60541 (8)	0.11510 (14)	0.64634 (3)	0.0460 (3)	
O3	0.61014 (9)	0.54911 (17)	0.82831 (3)	0.0535 (3)	
H3	0.5439	0.5709	0.8329	0.080*	
C1	0.86784 (12)	0.2014 (2)	0.51899 (5)	0.0407 (4)	
C2	0.79314 (13)	0.0874 (2)	0.49804 (5)	0.0487 (4)	
H2	0.7279	0.0556	0.5132	0.058*	
C3	0.81269 (14)	0.0203 (2)	0.45558 (5)	0.0535 (4)	
H3A	0.7616	−0.0568	0.4423	0.064*	
C4	0.90936 (15)	0.0683 (2)	0.43253 (5)	0.0518 (4)	
C5	0.98315 (15)	0.1872 (2)	0.45215 (6)	0.0567 (5)	
H5	1.0465	0.2232	0.4363	0.068*	
C6	0.96304 (14)	0.2522 (2)	0.49499 (6)	0.0498 (4)	
H6	1.0135	0.3308	0.5081	0.060*	
C7	0.8819 (2)	−0.1482 (3)	0.37488 (7)	0.0800 (6)	
H7A	0.8849	−0.2396	0.3975	0.120*	
H7B	0.9171	−0.1893	0.3476	0.120*	
H7C	0.8052	−0.1190	0.3687	0.120*	
C8	0.84886 (12)	0.2682 (2)	0.56475 (5)	0.0426 (4)	
H8	0.8944	0.3574	0.5763	0.051*	
C9	0.67022 (11)	0.23670 (19)	0.65719 (5)	0.0363 (3)	
C10	0.65784 (11)	0.33283 (19)	0.70077 (5)	0.0359 (3)	
C11	0.74372 (12)	0.3390 (2)	0.73227 (5)	0.0418 (4)	
H11	0.8139	0.2948	0.7247	0.050*	
C12	0.72609 (12)	0.4102 (2)	0.77481 (5)	0.0460 (4)	
H12	0.7839	0.4114	0.7960	0.055*	
C13	0.62246 (12)	0.4800 (2)	0.78614 (5)	0.0391 (4)	
C14	0.53678 (12)	0.4772 (2)	0.75443 (5)	0.0414 (4)	
H14	0.4675	0.5261	0.7615	0.050*	
C15	0.55430 (12)	0.4021 (2)	0.71250 (5)	0.0422 (4)	
H15	0.4958	0.3977	0.6917	0.051*	
H2A	0.8008 (14)	0.3787 (19)	0.6374 (6)	0.080*	

### Atomic displacement parameters (Å^2^)

**Table:**

	*U*^11^	*U*^22^	*U*^33^	*U*^12^	*U*^13^	*U*^23^
N1	0.0453 (7)	0.0455 (8)	0.0375 (7)	−0.0003 (6)	0.0089 (6)	−0.0040 (6)
N2	0.0450 (7)	0.0437 (7)	0.0393 (7)	−0.0044 (6)	0.0098 (6)	−0.0066 (6)
O1	0.1010 (11)	0.0751 (10)	0.0503 (8)	−0.0109 (8)	0.0234 (7)	−0.0152 (6)
O2	0.0436 (6)	0.0499 (7)	0.0447 (6)	−0.0074 (5)	0.0081 (5)	−0.0078 (5)
O3	0.0464 (6)	0.0737 (8)	0.0404 (6)	0.0078 (6)	0.0045 (5)	−0.0117 (5)
C1	0.0425 (8)	0.0401 (8)	0.0396 (8)	0.0018 (7)	0.0081 (6)	0.0033 (6)
C2	0.0471 (9)	0.0513 (10)	0.0478 (9)	−0.0039 (8)	0.0096 (7)	0.0015 (7)
C3	0.0601 (11)	0.0542 (10)	0.0464 (9)	−0.0064 (8)	0.0015 (8)	−0.0035 (8)
C4	0.0642 (11)	0.0501 (10)	0.0412 (9)	0.0023 (9)	0.0109 (8)	0.0001 (7)
C5	0.0597 (10)	0.0585 (11)	0.0519 (10)	−0.0062 (9)	0.0217 (8)	−0.0006 (8)
C6	0.0501 (9)	0.0482 (9)	0.0512 (9)	−0.0069 (8)	0.0115 (7)	−0.0013 (7)
C7	0.1133 (17)	0.0747 (14)	0.0520 (11)	−0.0059 (14)	0.0008 (11)	−0.0156 (10)
C8	0.0411 (8)	0.0418 (9)	0.0449 (8)	−0.0003 (7)	0.0058 (7)	0.0002 (7)
C9	0.0356 (7)	0.0365 (8)	0.0369 (8)	0.0022 (6)	0.0015 (6)	0.0018 (6)
C10	0.0359 (7)	0.0355 (8)	0.0363 (7)	−0.0006 (6)	0.0051 (6)	0.0011 (6)
C11	0.0320 (7)	0.0495 (9)	0.0438 (8)	0.0056 (7)	0.0050 (6)	−0.0003 (7)
C12	0.0364 (8)	0.0616 (11)	0.0401 (8)	0.0057 (7)	−0.0030 (6)	−0.0039 (7)
C13	0.0401 (8)	0.0411 (8)	0.0360 (8)	0.0000 (7)	0.0059 (6)	0.0005 (6)
C14	0.0319 (7)	0.0442 (9)	0.0480 (9)	0.0034 (6)	0.0056 (6)	−0.0051 (7)
C15	0.0339 (8)	0.0477 (9)	0.0449 (8)	0.0021 (7)	−0.0019 (6)	−0.0039 (7)

### Geometric parameters (Å, °)

**Table:**

N1—C8	1.2758 (18)	C5—H5	0.93
N1—N2	1.3850 (16)	C6—H6	0.93
N2—C9	1.3408 (18)	C7—H7A	0.96
N2—H2A	0.901 (9)	C7—H7B	0.96
O1—C4	1.3610 (19)	C7—H7C	0.96
O1—C7	1.418 (2)	C8—H8	0.93
O2—C9	1.2431 (17)	C9—C10	1.4822 (19)
O3—C13	1.3554 (17)	C10—C11	1.3841 (19)
O3—H3	0.82	C10—C15	1.3868 (19)
C1—C2	1.385 (2)	C11—C12	1.3797 (19)
C1—C6	1.393 (2)	C11—H11	0.93
C1—C8	1.457 (2)	C12—C13	1.386 (2)
C2—C3	1.370 (2)	C12—H12	0.93
C2—H2	0.93	C13—C14	1.386 (2)
C3—C4	1.388 (2)	C14—C15	1.375 (2)
C3—H3A	0.93	C14—H14	0.93
C4—C5	1.385 (2)	C15—H15	0.93
C5—C6	1.375 (2)		
			
C8—N1—N2	114.89 (13)	O1—C7—H7C	109.5
C9—N2—N1	118.86 (13)	H7A—C7—H7C	109.5
C9—N2—H2A	123.0 (12)	H7B—C7—H7C	109.5
N1—N2—H2A	117.5 (12)	N1—C8—C1	120.25 (14)
C4—O1—C7	117.85 (15)	N1—C8—H8	119.9
C13—O3—H3	109.5	C1—C8—H8	119.9
C2—C1—C6	118.11 (14)	O2—C9—N2	122.26 (13)
C2—C1—C8	121.81 (13)	O2—C9—C10	121.50 (12)
C6—C1—C8	120.08 (14)	N2—C9—C10	116.22 (13)
C3—C2—C1	121.80 (15)	C11—C10—C15	118.78 (13)
C3—C2—H2	119.1	C11—C10—C9	121.54 (12)
C1—C2—H2	119.1	C15—C10—C9	119.31 (13)
C2—C3—C4	119.46 (16)	C12—C11—C10	120.58 (13)
C2—C3—H3A	120.3	C12—C11—H11	119.7
C4—C3—H3A	120.3	C10—C11—H11	119.7
O1—C4—C5	116.26 (15)	C11—C12—C13	120.21 (13)
O1—C4—C3	124.07 (16)	C11—C12—H12	119.9
C5—C4—C3	119.67 (15)	C13—C12—H12	119.9
C6—C5—C4	120.22 (15)	O3—C13—C14	122.91 (13)
C6—C5—H5	119.9	O3—C13—C12	117.64 (13)
C4—C5—H5	119.9	C14—C13—C12	119.44 (13)
C5—C6—C1	120.67 (16)	C15—C14—C13	119.94 (13)
C5—C6—H6	119.7	C15—C14—H14	120.0
C1—C6—H6	119.7	C13—C14—H14	120.0
O1—C7—H7A	109.5	C14—C15—C10	121.01 (13)
O1—C7—H7B	109.5	C14—C15—H15	119.5
H7A—C7—H7B	109.5	C10—C15—H15	119.5

### Hydrogen-bond geometry (Å, °)

**Table:**

*D*—H···*A*	*D*—H	H···*A*	*D*···*A*	*D*—H···*A*
O3—H3···O2^i^	0.82	1.91	2.7271 (15)	171
N2—H2A···O2^ii^	0.90 (1)	2.12 (1)	3.0216 (17)	173 (2)

Symmetry codes: (i) −*x*+1, *y*+1/2, −*z*+3/2; (ii) −*x*+3/2, *y*+1/2, *z*.
